# Characterization of the MicroRNA profile in rheumatoid arthritis plasma exosomes and their roles in B-cell responses

**DOI:** 10.1016/j.clinsp.2024.100441

**Published:** 2024-07-07

**Authors:** Jian Lu, Jing Wu, Xiao Zhang, Rui Zhong, BingYing Wang, Huan Yang, Ping Feng

**Affiliations:** a Department of Clinical Laboratory, The Second Affiliated Hospital of Soochow University, Suzhou, Jiangsu, China; b Department of Laboratory Medicine, the Affiliated Guangji Hospital of Soochow University, Suzhou Mental Health Center, Suzhou, Jiangsu, China; c Department of Orthopedics, The Second Affiliated Hospital of Soochow University, Suzhou, Jiangsu, China

**Keywords:** Rheumatoid Arthritis, Plasma, Exosomes, MicroRNAs

## Abstract

**Objective:**

This study aimed to identify differentially expressed microRNAs (miRNAs) in exosomes derived from the blood plasma of Rheumatoid Arthritis (RA) patients and explore their clinical significance and biological roles.

**Methods:**

Illumina high-throughput sequencing was employed to measure miRNA expression levels in plasma exosomes, followed by validation using qRT-PCR. The correlation between exosomal miRNAs and disease activity was systematically analyzed. Additionally, the pathogenic effects of RA exosomes were investigated through bioinformatics analysis and in vitro experiments.

**Results:**

Significantly reduced levels of exosomal miR-144-3p and miR-30b-5p were observed in RA patients, which were negatively correlated with DAS28 scores and anti-CCP antibody levels. ROC curve analysis showed that miR-144-3p and miR-30b-5p in plasma exosomes could effectively distinguish RA patients from healthy controls, with AUC values of 0.725 and 0.773, respectively. Combining bioinformatics analysis and in vitro experiments, it was demonstrated that plasma exosomes contribute to ongoing autoantibody production in RA by promoting B-cell differentiation and antibody production.

**Conclusion:**

The present study indicates that plasma exosomes from RA patients may be potentially pathogenic. Exosomal miR-144-3p and miR-30b-5p exhibit significant decreases in RA patients and are associated with disease activity, suggesting their potential as valuable biomarkers for RA.

## Highlights

• The levels of plasma exosomal miR-144-3p and miR-30b-5p are significantly decreased in RA patients, which are negatively correlated with RA disease activity.
• Plasma exosomes from RA patients are pathogenic and may be involved in disease progression by enhancing B-cell differentiation and antibody production.
• Plasma exosomal miR-144-3p and miR-30b-5p are potential serologic markers for RA.

## Introduction

Rheumatoid Arthritis (RA) manifests as inflammation and cartilage destruction in affected joints, resulting in joint dysfunction and deformity.^1^^,^^2^ By the time patients seek medical attention, they often typically find themselves in the middle or advanced stages of the disease. Although conventional drugs can slow disease progression and alleviate symptoms, they do not provide a cure. Consequently, early detection and precise disease monitoring are crucial, highlighting the need for more effective diagnostic indicators in clinical settings.

Exosomes are small vesicles released by cells that selectively transport protein and nucleic acid molecules, including mRNA, lncRNA, and miRNA, from their cells of origin. These molecules are crucial for intercellular signaling.^3^^,^^4^ In the context of RA, exosomes demonstrate pathogenic effects.^5^ For example, exosomes secreted by synovial fibroblasts transport miR-424, which targets FOXP3 and consequently promotes the differentiation of Th17 cells while inhibiting Treg cell production.^6^ Additionally, exosomes from Peripheral Blood Mononuclear Cells (PBMCs) may exacerbate RA progression by delivering the long non-coding RNA (lncRNA) NEAT1.^7^ Consequently, investigating alterations in exosomes among RA patients could elucidate the disease's underlying mechanisms. Notably, the functional molecules encapsulated within exosomes are protected by a double membrane structure, enhancing their stability and resistance to degradation. This characteristic underscores the potential of exosomes as disease markers for clinical use.^8^^,^^9^

This study aimed to investigate the differential expression of miRNAs in plasma exosomes of RA patients and to explore their potential clinical implications. Additionally, the authors conducted bioinformatics analyses of miR-144-3p and miR-30b-5p in plasma exosomes, which included target gene prediction, GO enrichment analysis, and KEGG enrichment analysis. Furthermore, the authors preliminarily explored the pathogenic role of plasma exosomes through in vitro experiments. The objective of this study was to identify serological markers with promising clinical utility and to preliminarily elucidate their potential roles, thereby aiming to offer new targets and insights for the clinical diagnosis and treatment of RA.

## Materials and methods

### Study subjects

The study included patients diagnosed with Rheumatoid Arthritis (RA) who were treated at the Department of Rheumatology and Immunology at the Second Affiliated Hospital of Soochow University from January 1 to July 7, 2023, forming the experimental group. The control group comprised healthy individuals who underwent a physical examination during the same timeframe. Inclusion criteria for RA patients were adherence to the 2009 diagnostic criteria for rheumatoid arthritis as revised by the American College of Rheumatology, absence of previous medical conditions, presence of an active disease stage (DAS28-CRP ≥ 2.6), and absence of concurrent autoimmune disorders. Healthy controls were required to meet criteria such as normal blood counts, healthy liver and kidney functions, negative results on quantitative Anti-Nuclear Antibody (ANA) tests, and no history of autoimmune disease. The study obtained approval from the ethics committee of the Second Affiliated Hospital of Soochow University, and all participants provided written informed consent.

### Sample collection and exosome isolation

Fasting venous blood (5 mL, anticoagulated with EDTA-K2) was collected, and plasma was then separated by centrifugation at 3,000 rpm and 4 °C for 10 min. The plasma was subjected to a second centrifugation in an RNAase-free tube (3,000g, 4 °C for 15 min) to adequately remove cellular debris. The supernatant was then subjected to a third centrifugation (12,000g, 4 °C for 10 min) followed by filtration through a 0.22 μm filter. A mixture of 400 μL of plasma and 100 μL of Exosome Isolation Reagent (SBI, USA) was incubated at 4 °C for 30 min following thorough shaking. The supernatant was discarded through centrifugation at 1,500g for 30 min at 4 °C, thus isolating the exosome precipitate. The protein concentration of the exosomes was measured using a BCA protein quantification kit (ComWin Biotech, China). The particle size distribution was determined using a nanoparticle tracking analyzer (Particle Metrix, Germany), and the morphology of exosomes was observed by transmission electron microscopy (Tecnai-12, Philips, Netherlands).

### Western blot

Proteins extracted from exosomes/cell lysates were separated using 12 % SDS-PAGE. The proteins were then transferred to PVDF membranes (Merck, USA), which were subsequently blocked with 5 % BSA in TBST and incubated with CD63, TSG101, and Calnexin antibodies (Abcam, England). Following incubation with HRP-conjugated secondary antibodies, the signals were detected using the ECL method and a gel imager system (GE Healthcare, USA).

### Exosomal small RNA high-throughput sequencing

Plasma samples were obtained from nine patients diagnosed with active-phase Rheumatoid Arthritis (RA). These samples were divided into three groups, with each group comprising three randomly selected cases. Likewise, nine samples from healthy individuals were also grouped in the same manner to serve as controls. Total RNA was extracted from plasma exosomes using the Trizol method. Small RNA libraries were constructed and sequenced on the Illumina sequencing platform (HiSeqTM 2500, San Diego, California, USA) to identify differentially expressed miRNAs.

### qRT-PCR

Exosomal RNA was extracted from both the RA and healthy control groups using Trizol. Following poly (A) tailing, the RNA was reverse-transcribed into complementary DNA (cDNA). Quantitative PCR amplification was conducted using SYBR Green in a 20 μL reaction system under the following conditions: 95 °C for 10 min, followed by 40 cycles of 95 °C for 5 s, 60 °C for 20 s, and 70 °C for 10 s. RNU6 was utilized as the internal reference gene, and the results were analyzed using the 2^−ΔΔCt^ method. The primer sequences utilized for RT-qPCR are listed in Table 1.

**Table 1:** Primer sequence of 5 miRNAs.

Primer name	Primer sequence (5′ to 3′)
hsa-miR-20a-5p	GCGTAAAGTGCTTATAGTGCAGGTAG
hsa-miR-30b-5p	GCTGTAAACATCCTACACTCAGCT
hsa-miR-144-3p	CGGCCGCTACAGTATAGATGATGT
hsa-miR-223-5p	GCCGTGTATTTGACAAGCTGAGTT
hsa-miR-589-5p	TGAGAACCACGTCTGCTCTGAG

### KEGG and GO enrichment analysis of miRNA target genes

The target genes of miRNAs were predicted utilizing TargetScan, miRDB, miRTarBase, and miRWalk, with a minimum requirement of two databases to predict the same target gene. A significance threshold of *p* < 0.05 was established for the Gene Ontology (GO) enrichment analysis, which aimed to identify the Molecular Function (MF), Biological Process (BP), and Cellular Component (CC) associated with the target genes. The Kyoto Encyclopedia of Genes and Genomes (KEGG) enrichment analysis was employed to identify signal transduction and disease pathways.

### B cells in vitro culture and flow cytometry analysis

Peripheral Blood Mononuclear Cells (PBMCs) were extracted from the peripheral blood of RA patients under aseptic conditions using density gradient centrifugation, and total B-cells were isolated with CD19 MicroBeads (Miltenyi Biotec, Germany) through magnetically activated cell sorting (MACS). Subsequently, 50 μg of plasma exosomes were used to treat 5 × 10^5^ B-cells in conjunction with 5 μg/mL anti-IgM mAb at 37 °C for 72 h. B-cells were subsequently collected and blocked with Fc Block (Miltenyi Biotec, Germany) at 4 °C for 10 min. Surface markers were stained with anti-human CD19 and CD138 fluorescent-conjugated antibodies (eBioscience, USA) and gently incubated for 45 min. After being washed twice with PBS, cells were resuspended in 200 μL of PBS and analyzed using a BD canto II cytometry.

### Enzyme-linked immunosorbent assay

Total IgM and IgG levels were assessed following the manufacturer's instructions (MultiSciences, China). Briefly, 100 μL of diluted culture supernatant was added to the ELISA plate and incubated for 60 min at 37 °C, followed by the addition of HRP-conjugated antibodies at room temperature for another 60 min. Subsequently, 100 μL of TMB was added and incubated for 15 min at 37 °C, followed by the termination of the reaction with H_2_SO_4_. The Optical Density (OD) values of the samples were determined at a wavelength of 450 nm using a microplate reader.

### Statistical analysis

Statistical significance was assessed using the Student's *t*-test or one-way Analysis of Variance (ANOVA), and the data were presented as mean ± SEM. Correlations were evaluated using the Spearman correlation coefficient. The Receiver Operating Characteristic (ROC) curve was utilized to assess the accuracy of plasma exosomal miRNAs in discriminating between Rheumatoid Arthritis (RA) patients and Healthy Controls (HC), and the test accuracy was calculated using the Area Under the Curve (AUC). All analyses were conducted using Prism version 6.02 (GraphPad Software, Inc., San Diego, CA, USA), with statistical significance set at *p* < 0.05.

## Results

### RA exosomes preparation and miRNA sequencing

**Fig. 1:**
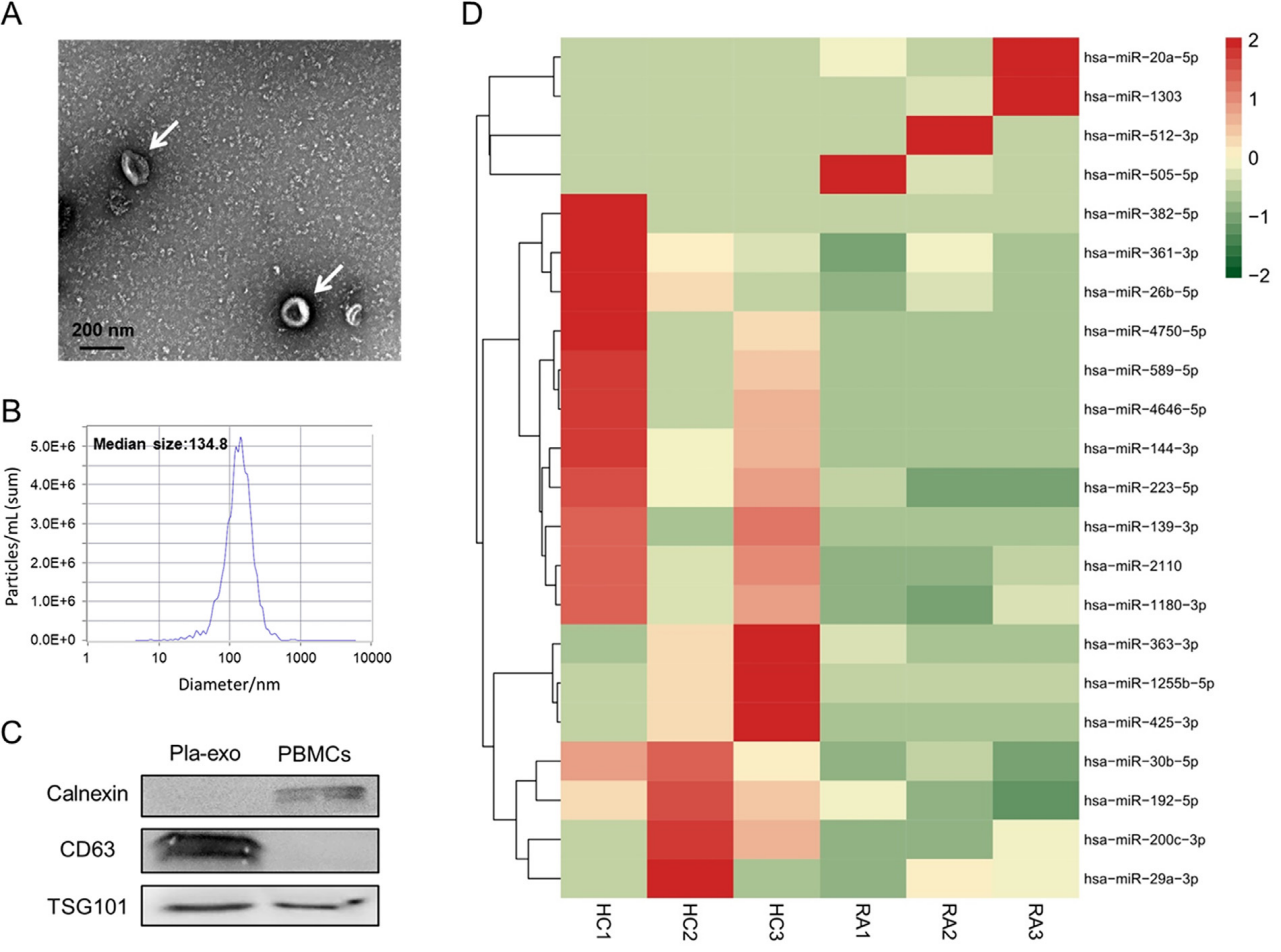
Preparation and small RNA sequencing of plasma exosomes from RA patients. (A) Morphology of plasma exosomes (from RA patients) observed under electron microscopy. (B) Nanoparticle tracking analysis of plasma exosomes (from RA patients). (C) Western blot analysis of characteristic proteins of plasma exosomes (from RA patients) using the indicated monoclonal antibodies. (D) Heatmap plots illustrating differentially expressed miRNAs between RA patients and healthy controls.

Plasma-derived exosomes from Rheumatoid Arthritis (RA) patients displayed a disc-like structure when observed under electron microscopy (Fig. 1A), with particle sizes ranging between 50 to 150 nm (Fig. 1B). As shown in Fig. 1C, exosome-associated proteins, including CD63 and TSG101, were found in abundance, while apoptotic/necrosis markers, such as calnexin, were absent in exosomes as determined by western blot analysis. High-throughput sequencing was utilized to identify the abnormally expressed miRNAs in plasma exosomes. Sequencing identified 22 differentially expressed exosomal miRNAs between RA patients and healthy donors, with a screening criterion of a p-value less than 0.05. Among these, 4 were up-regulated, and 18 were down-regulated in RA patients (Fig. 1D). Considering both the significance of the p-values and the changes in content, five miRNAs (miR-144-3p, miR-30b-5p, miR-20a-5p, miR-223-5p, and miR-589-5p) were initially identified as potentially associated with RA.

### The differentially expressed miRNAs in RA exosomes

**Fig. 2:**
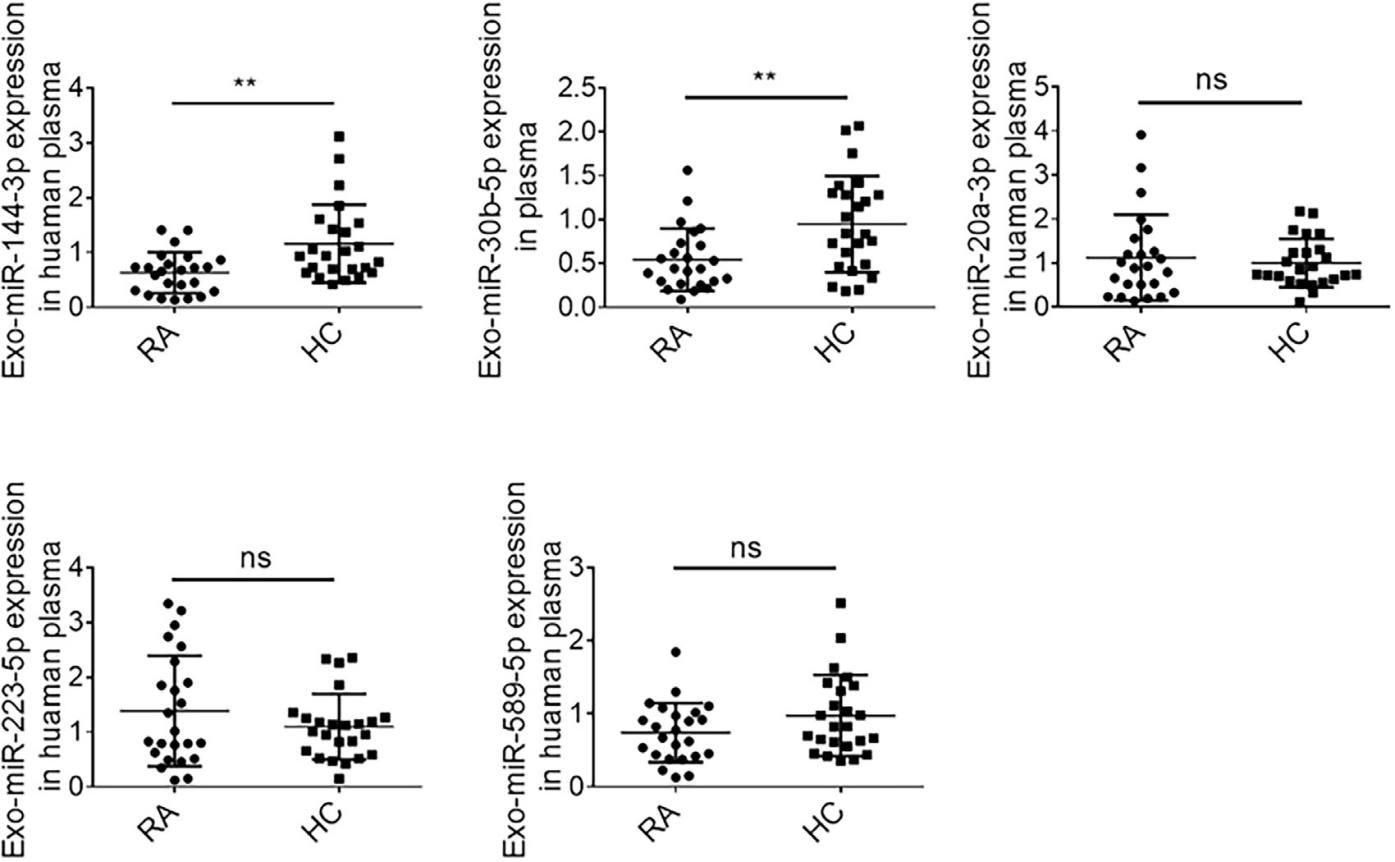
Differential expression of miRNAs in RA exosomes. The levels of plasma exosomal miR-144-3p, miR-30b-5p, miR-20a-5p, miR-223-5p, and miR-589-5p in RA patients (n = 24) and Healthy Controls (HC) (*n* = 24) were quantified by qRT-PCR. Data are presented as mean ± SEM (**p < 0.01).

Plasma samples were collected from RA patients and healthy controls, with the clinical features of both groups detailed in Table 2. Exosomal RNA was extracted, and the levels of miR-144-3p, miR-30b-5p, miR-20a-5p, miR-223-5p, and miR-589-5p in the exosomes were quantified via qRT-PCR. The results demonstrated that the levels of miR-144-3p (*p* < 0.05) and miR-30b-5p (*p* < 0.05) in plasma exosomes were significantly lower in active RA patients than in the controls (Fig. 2). However, the expression levels of miR-20a-5p, miR-223-5p, and miR-589-5p did not show statistically significant differences.

**Table 2:** Clinical, demographic, and serological characteristics of RA patients and HC.

	RA patients (n = 24)	HC (n = 24)
**Demographic parameters**		
Female/male	(19/5)	(18/6)
Age, years (median [IQR])	59 (31‒79)	60 (38‒77)
**Disease activity**		
DAS28 (mean ± SD)	5.38 ± 0.44	
**Laboratory parameters**		
RF, IU/mL	220.30 ± 54.09	
Anti-CCP, RU/mL	293.40 ± 90.96	
ESR, mm/h (mean ± SD)	34.36 ± 4.33	
CRP, mg/dL (mean ± SD)	26.68 ± 9.41	

DAS28, Disease Activity Score-28; RF, Rheumatoid Factor; Anti-CCP, Anti-Cyclic Citrullinated Peptide; ESR, Erythrocyte Sedimentation Rate; CRP, C-Reactive Protein.

### Associations between exosomal miRNAs and clinical characteristics of RA

**Fig. 3:**
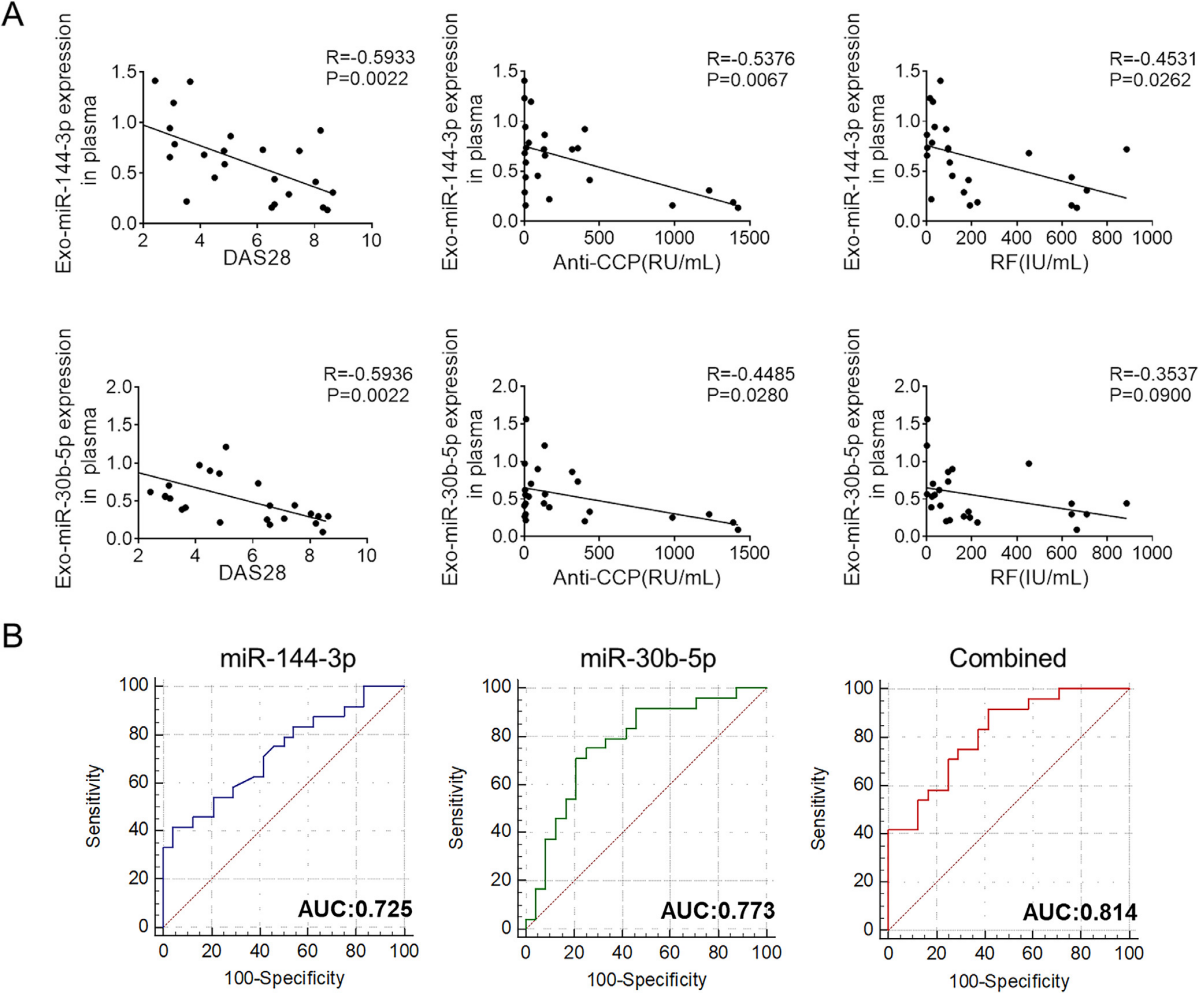
Associations between exosomal miRNAs and clinical characteristics of RA. (A) Correlation analysis of plasma exosomal-miR-144-3p and miR-30b-5p with DAS28 scores and RF and anti-CCP antibody levels in RA patients (*n* = 24). Data were analyzed using Spearman's rank correlation test (*p < 0.01, **p < 0.01). (B) A ROC curve was utilized to evaluate the accuracy of plasma exosomal miR-144-3p/miR-30b-5p in differentiating between patients with RA (*n* = 24) and healthy controls (*n* = 24).

Recent studies have identified circulating exosomes as potential biomarkers.^10^^,^^11^ The authors hypothesized an association between exosomal miR-144-3p, miR-30b-5p, and the activity of RA. As depicted in Fig. 3A, a negative correlation was observed between the levels of plasma exosomal miR-144-3p, miR-30b-5p, and the DAS28 scores, as well as anti-CCP antibody levels in RA patients. Furthermore, the Area Under the ROC Curve (AUC) for both miR-144-3p and miR-30b-5p exceeded 0.7, indicating substantial diagnostic potential. The combined utilization of these two miRNAs significantly enhanced the diagnostic efficacy, with an AUC of 0.814 (95 % CI: 0.676‒0.912, *p* < 0.0001) (Fig. 3B), indicating their potential value in RA diagnosis.

### Bioinformatics analysis of ***miR-144-3p and miR-30b-5p***

**Fig. 4:**
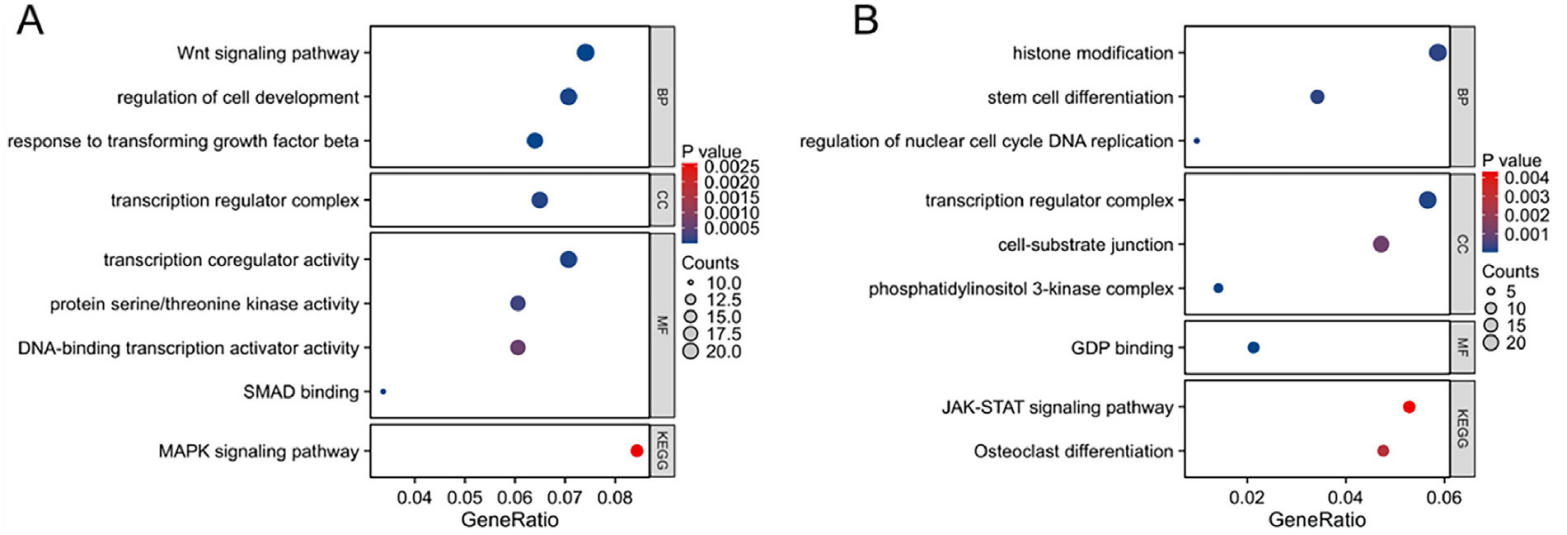
Bioinformatics analysis of the genes targeted by miR-144-3p and miR-30b-5p. GO and KEGG enrichment analyses of target genes for miR-144-3p (A) and miR-30b-5p (B) in Biological Process (BP), Cellular Components (CC), and Molecular Function (MF).

To further explore the pathogenic effects of plasma exosome-related miRNAs, the authors conducted comprehensive bioinformatics analyses of miR-144-3p and miR-30b-5p. Target gene prediction and bioinformatic analysis of miR-144-3p and miR-30b-5p were performed using four databases: miRDB, miRWalk, TargetScan, and miRTarBase. GO and KEGG enrichment analyses revealed that the genes targeted by miR-144-3p predominantly involved in signaling pathways, such as TGF-β, Wnt, and MAPK, which play crucial roles in cell differentiation and development, transcriptional regulation, and serine/chromosome activity (Fig. 4A). Conversely, genes targeted by miR-30b-5p are primarily involved in PI3K and JAK-STAT signaling pathways, which regulate key biological processes including histone modification, cell cycle, and osteoblast differentiation (Fig. 4B). These results suggest that changes in the levels of miR-144-3p and miR-30b-5p in RA exosomes may be associated with the proliferation and differentiation of inflammation-associated immune cells.

### *RA exosomes induce B-cell differentiation and antibody production*

**Fig. 5:**
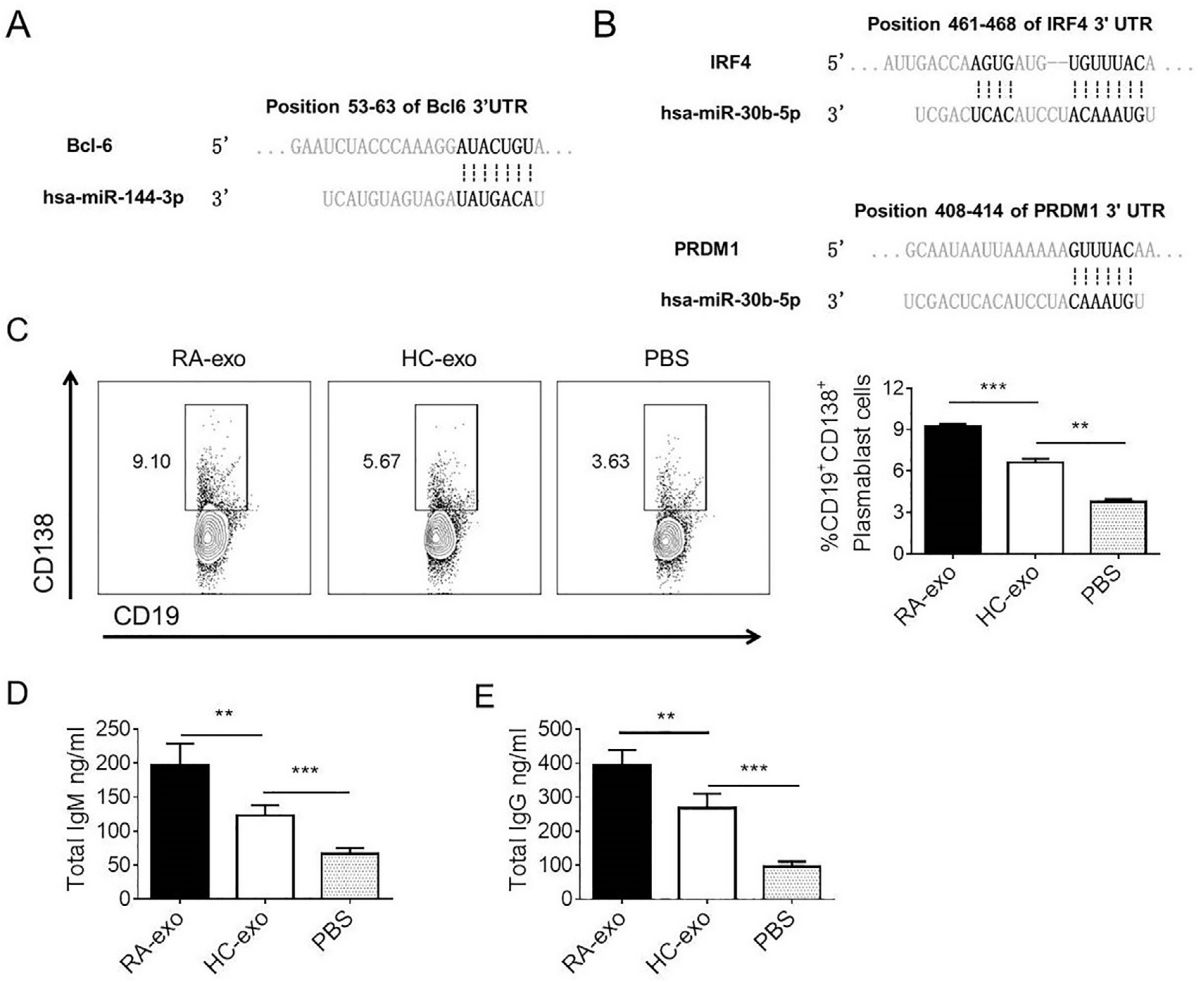
RA exosomes induce B-cell differentiation and antibody production. (A) The putative miR-144-3p-binding sites (position 53‒63) on the 3’UTR of the BCL6 mRNA, with potential complementary residues highlighted in bold, are shown. (B) The putative miR-30b-5p-binding sites (position 461‒468) on the 3’UTR of the IRF4 mRNA, and the putative miR-30b-5p-binding sites (positions 408‒414) on the 3′UTR of PRDM1 mRNA, with potential complementary residues highlighted in bold. (C) A total of 5×10^5^ B-cells isolated from RA-PBMCs were incubated with 50 μg exosomes at 37 °C for 4 days and then incubated with fluorescent-conjugated anti-CD19 and CD138 antibodies. The PBS-treated group was used as a negative control. The data were analyzed by Flow Cytometry (FCM). The total IgM (D) and IgG (E) in the supernatant were measured by ELISAs. **p < 0.01, ***p < 0.001. The data are from three independent experiments (mean ± SEM) or are representative of three independent experiments.

As an autoimmune disease, RA is characterized by B-cell overactivation accompanied by the high production of pathogenic antibodies.^12^ Given the robust correlation between exosome-derived miR-144-3p and miR-30b-5p and autoantibody levels in RA patients, the authors hypothesized that exosomes containing these miRNAs might contribute to disease progression by modulating autoantibody production. In support of this hypothesis, the target gene prediction results identified IRF-4/Blimp-1 and Bcl-6 as targets for miR-30b-5p and miR-144-3p, respectively (Fig. 5A and 5B). Considering the crucial roles of IRF-4/Blimp-1 and Bcl-6 in plasma cell and Tfh cell differentiation, it is conceivable that RA plasma exosomes may influence disease progression by regulating autoantibody production. Thus, the authors conducted further investigations into the role of RA exosomes in the differentiation and antibody production of B-cells. As shown in Figs. 5C, 5D, and 5E, compared to exosomes from Healthy individuals (HC-exo), RA exosomes (RA-exo) significantly induced B-cell differentiation into plasmablast cells and promoted IgM and IgG production. These findings suggest that plasma exosomes from RA patients are associated with antibody secretion and could potentially contribute to RA disease progression.

## Discussion

Exosomes, membranous vesicles, selectively encapsulate biomolecules from parent cells, exhibiting superior stability in blood compared to traditional serological indicators. This characteristic positions exosomes as novel biomarkers with significant clinical implications.^4^^,^^13^ Accordingly, the focus on biomarker discovery has centered on miRNAs within plasma exosomes, given their potential as serological markers and their ability to regulate the expression of relevant pathogenic genes.^14^^,^^15^ Plasma-free miRNA levels have demonstrated a notable correlation with RA disease activity and prognosis. Several studies have reported significantly elevated plasma miR-155 levels in RA patients compared to healthy controls. However, findings on miR-155 levels in RA patient plasma have displayed inconsistency, with some studies reporting decreased levels or no change. Similarly, findings regarding changes in miR-22 levels in RA patient plasma have shown variation across studies.^16^ These discrepancies could stem from factors such as miRNA analysis techniques, study design, and disease stage. The instability and short half-life of circulating miRNAs in peripheral blood may also contribute to these inconsistencies. Conversely, exosomal miRNAs, which are shielded by the double membrane structure, exhibit stability and possess a longer half-life,^17^ rendering them promising disease markers for clinical applications.

In this study, the authors identified 22 miRNAs exhibiting abnormal expression in the plasma exosomes of patients with active Rheumatoid Arthritis (RA). Among these, the focus was on five differentially expressed miRNAs: miR-144-3p, miR-30b-5p, miR-20a-5p, miR-223-5p, and miR-589-5p, which the authors validated using qRT-PCR. The present results revealed significant decreases in the levels of miR-144-3p and miR-30b-5p in the plasma exosomes of RA patients. These reductions were negatively correlated with the disease activity score DAS28 and anti-CCP antibody levels. Additionally, ROC curve analysis demonstrated that exosome-derived miR-144-3p and miR-30b-5p effectively distinguished RA patients from Healthy Controls (HC), supporting the possibility that plasma exosomal miR-144-3p and miR-30b-5p are potential biomarkers for RA.

Bioinformatics analysis indicated that miR-144-3p could influence cell differentiation and development by modulating TGF-β, Wnt, and MAPK signaling pathways. Conversely, miR-30b-5p may regulate cell proliferation and differentiation by affecting PI3K and JAK-STAT signaling. Given the strong correlation between the levels of plasma exosomal miR-144-3p and miR-30b-5p and anti-CCP antibodies, the authors utilized gene prediction software to investigate their potential interactions with transcription factors associated with Tfh/plasma cell differentiation. The present findings revealed that Bcl-6 and IRF-4/Blimp-1 are target genes for miR-144-3p and miR-30b-5p, respectively. Interestingly, in vitro experiments showed that RA exosomes significantly enhance plasmablast differentiation and antibody production. All these results indicate that plasma exosomes from RA patients are pathogenic and could be involved in disease progression and auto-antibody production.

## Conclusion

In summary, this study primarily focused on comparing the diversity of plasma-derived exosomes between individuals with active Rheumatoid Arthritis (RA) and healthy individuals. However, analyses based on small-scale samples may yield unreliable results, and accurate data comparisons necessitate large-scale prospective cohort studies. Additionally, this study focused solely on comparing the heterogeneity of plasma-derived exosomes between RA active phase patients and healthy controls. The future evaluation of exosomal miRNAs as serological markers for RA disease must incorporate relevant data from patients in remission and at different stages of disease onset. Despite being in its early stages, the study of the biological functions and clinical applications of exosomal miRNAs encounters several challenges. These include the establishment of efficient methods and standardized protocols for exosome extraction, gaining a deeper understanding of the biological functions and specific mechanisms of action of exosomal miRNAs, and the development of more sensitive and reliable detection methods for exosomal miRNAs in plasma. However, with advancements in exosome research and related detection technologies, the use of exosome-related markers as novel serological indicators for RA is becoming increasingly feasible.^18^^,^^19^ In conclusion, by employing high-throughput sequencing technology, the authors identified a set of differentially expressed miRNAs in the plasma exosomes of RA patients, thus establishing the association between plasma exosomal miR-144-3p and miR-30b-5p with disease activity and their potential as serological markers for RA.

### Abbreviations

miR, microRNA; RA, Rheumatoid Arthritis; DAS28, Disease Activity Score-28; CCP, Cyclic Citrullinated Peptide; RF, Rheumatoid Factor; ESR, Erythrocyte Sedimentation Rate; CRP, C-Reactive Protein.

## Ethical approval

This study was carried out in accordance with the Declaration of Helsinki and was approved by the ethics committee of the Second Affiliated Hospital of Soochow University (JD-LK2023125-I01). Signed informed consent forms were obtained from each individual. All experiments complied with the ARRIVE guidelines.

## Consent to publish

The present manuscript did not contain any individual details, images, or videos. The authors used to number the cases to maintain the confidentiality of patient data.

## Authors’ contributions

JL conceptualized the study, analyzed the data, and wrote the manuscript; JW, XZ, RZ, and BY W performed the experiments; PF and HY discussed and revised the manuscript. All authors read and approved the final manuscript.

## Funding

This work was supported by the Project of Medical Research Fund of Jiangsu Provincial Health Commission (Z2023043), Gusu Talent Program (2023) 024, The Project of Suzhou Science and Technology Bureau (SKY2023228), Advance Research Program of The Second Affiliated Hospital of Soochow University (SDFEYBS2203).

## Declaration of competing interest

The authors declare no conflicts of interest.

## References

[1] Yang J, Li Q. (2023). Rheumatoid arthritis. N Engl J Med.

[2] Gravallese EM, Firestein GS. (2023). Rheumatoid arthritis ‒ common origins, divergent mechanisms. N Engl J Med.

[3] Logozzi M, Di Raimo R, Mizzoni D, Fais S. (2022). What we know on the potential use of exosomes for nanodelivery. Semin Cancer Biol.

[4] Kalluri R, LeBleu VS. (2020). The biology, function, and biomedical applications of exosomes. Science.

[5] Fang Y, Ni J, Wang YS, Zhao Y, Jiang LQ, Chen C (2023). Exosomes as biomarkers and therapeutic delivery for autoimmune diseases: opportunities and challenges. Autoimmun Rev.

[6] Ding Y, Wang L, Wu H, Zhao Q, Wu S. (2020). Exosomes derived from synovial fibroblasts under hypoxia aggravate rheumatoid arthritis by regulating Treg/Th17 balance. Exp Biol Med (Maywood).

[7] Liu R, Jiang C, Li J, Li X, Zhao L, Yun H (2021). Serum-derived exosomes containing NEAT1 promote the occurrence of rheumatoid arthritis through regulation of miR-144-3p/ROCK2 axis. Ther Adv Chronic Dis.

[8] Liu J, Ren L, Li S, Li W, Zheng X, Yang Y (2021). The biology, function, and applications of exosomes in cancer. Acta Pharm Sin B.

[9] LeBleu VS, Kalluri R. (2020). Exosomes as a multicomponent biomarker platform in cancer. Trends Cancer.

[10] Mori MA, Ludwig RG, Garcia-Martin R, Brandão BB, Kahn CR. (2019). Extracellular miRNAs: from biomarkers to mediators of physiology and disease. Cell Metab.

[11] Xie F, Zhou X, Fang M, Li H, Su P, Tu Y (2019). Extracellular vesicles in cancer immune microenvironment and cancer immunotherapy. Adv Sci (Weinh).

[12] Neys SFH, Heutz JW, van Hulst JAC, Vink M, Bergen IM, de Jong PHP (2024). Aberrant B-cell receptor signaling in circulating naïve and IgA(+) memory B-cells from newly-diagnosed autoantibody-positive rheumatoid arthritis patients. J Autoimmun.

[13] Pegtel DM, Gould SJ (2019). Exosomes. Annu Rev Biochem.

[14] Mirzaei R, Zamani F, Hajibaba M, Rasouli-Saravani A, Noroozbeygi M, Gorgani M (2021). The pathogenic, therapeutic and diagnostic role of exosomal microRNA in the autoimmune diseases. J Neuroimmunol.

[15] Suh JH, Joo HS, Hong EB, Lee HJ, Lee JM. (2021). Therapeutic application of exosomes in inflammatory diseases. Int J Mol Sci.

[16] Peng X, Wang Q, Li W, Ge G, Peng J, Xu Y (2023). Comprehensive overview of microRNA function in rheumatoid arthritis. Bone Res.

[17] Song Q, Yu H, Han J, Lv J, Lv Q, Yang H. (2022). Exosomes in urological diseases - Biological functions and clinical applications. Cancer Lett.

[18] Yang D, Zhang W, Zhang H, Zhang F, Chen L, Ma L (2020). Progress, opportunity, and perspective on exosome isolation - efforts for efficient exosome-based theranostics. Theranostics.

[19] Lai JJ, Chau ZL, Chen SY, Hill JJ, Korpany KV, Liang NW (2022). Exosome processing and characterization approaches for research and technology development. Adv Sci (Weinh).

